# Sentinel Lymph Node Biopsy in Uterine Cervical Cancer Patients: Ready for Clinical Use? A Review of the Literature

**DOI:** 10.1155/2014/841618

**Published:** 2014-01-16

**Authors:** Viktoria-Varvara Palla, Georgios Karaolanis, Demetrios Moris, Aristides Antsaklis

**Affiliations:** ^1^1st Department of Obstetrics and Gynecology, School of Medicine, National & Kapodistrian University of Athens, “Alexandra” General Hospital, Athens, Greece; ^2^2nd Department of Surgery, Vascular Surgery Unit, Laiko General Hospital, Medical School of Athens, Athens, Greece; ^3^1st Department of Surgery, Vascular Surgery Unit, Laiko General Hospital, Medical School of Athens, Athens, Greece

## Abstract

Sentinel lymph node biopsy has been widely studied in a number of cancer types. As far as cervical cancer is concerned, this technique has already been used, revealing both positive results and several issues to be solved. The debate on the role of sentinel lymph node biopsy in cervical cancer is still open although most of the studies have already revealed its superiority over complete lymphadenectomy and the best handling possible of the emerging practical problems. Further research should be made in order to standardize this method and include it in the clinical routine.

## 1. Introduction

Cervical carcinoma is the commonest gynaecological cancer worldwide with almost 500.000 new cases per year and is particularly prevalent in the developing countries [1]. It is the tenth most common cancer affecting women in the developed countries [2]. The number of young women with cervical cancer has increased in recent years [3]. Thus, the effective use of screening has led to a rising number of women with cervical cancer being diagnosed in an early stage of the disease. Therefore, such patients must survive with treatment-associated sequelae for a long time and, in this way, prevention of some of these sequelae is important for this population. Lymph node metastasis is a central phenomenon in the natural history of patients with cervical cancer. The International Federation of Gynecology and Obstetrics (FIGO) staging system does not include lymph node status, but lymph node metastasis remains the most important risk factor for recurrence and death in surgically treated patients with early cervical cancer. The sentinel lymph node (SLN) is the first node draining the lymphatic flow from a primary tumor and represents the status of lymphatic spread [4, 5]. Therefore, if the sentinel node is negative, the remainder of the lymph nodes in the nodal basin should be free of disease as well, and it would not be reasonable to perform complete lymphadenectomy in case of negative sentinel lymph node. Cervical cancer is a good candidate disease for lymphatic mapping because of the following. Firstly, cervix has a complex lymphatic drainage due to its midline position. Secondly, conventional imaging techniques fail to identify with accuracy lymph node metastases. Thirdly, the incidence of nodal metastases in patients with tumor size less than or equal to 2 cm is 0–16% and in patients with stage IB is 15–31%. This means that a great number of patients with negative nodes will derive no benefit from lymphadenectomy [6]. On the contrary, they suffer from the possible side-effects of this procedure such as lymphoedema (10–15%), lymphocyst formation (up to 20%), neurovascular or ureteral injury, venous thromboembolism [2], infection, increased blood loss secondary to the dissection, and increased operative time [7–9]. Another benefit could be the increased detection of lymph node metastases through ultrastaging (up to 25% increase in metastases detection rate attributed to ultrastaging and identification of micrometastases) [10, 11] or removal of sentinel lymph nodes in aberrant locations or by ensuring complete removal of sentinel lymph nodes at the time of lymphadenectomy [12, 13]. In addition to the above, even if lymph node metastases are detected, patients can avoid being submitted to two treatment modalities, which are radical surgery and chemoradiation therapy. This option is really important for young women, who wish to preserve reproductive potential and could be treated with fertility-sparing radical trachelectomy [14]. Taking into consideration the facts above, the scientific world has introduced the possible clinical use of sentinel lymph node biopsy in cervical cancer patients.

## 2. History

It has been almost 100 years ago that Sappey published a study under the title “Anatomy, physiology and pathology of the lymphatic vessels in man and vertebrates,” where he injected Hg in dead body's skin and mapped the skin lymphatic drainage. In 1953, Sherman and Ter-Pogossian confirmed Sappey's statement that lymphatic drainage takes place in a predictable and regular manner. In 1977, Cabanas recognized the first node in the lymphatic basin in a case of penis neoplasm and introduced the term “sentinel lymph node.” Cabanas' pioneering study prompted other scientists to study the application of this method in a variety of cancer types [15]. In 1992, Morton et al. announced the first results of their study on lymphatic mapping in case of melanoma, through the injection of dye in the region of the lesion and the recognition, in 194 cases, of the sentinel lymph node. The percentage of metastases in sentinel lymph nodes was 21%, whereas in the rest of the nodes of the lymphatic basin, the percentage was much smaller (2 infiltrated lymph nodes out of 3000 nodes examined) [16]. In 1993, the lymphatic mapping of melanoma through radioisotopes injection and its signal detection were first described [17]. This method is easier and demands not so much experienced doctors; the node detection needs not so extensive dissection and its false-negative results are less. Finally, a combination of the two methods has come to practice, because of the greater accuracy and the less false-negative results accomplished.

## 3. Mapping Methods

As mentioned above, there are two main methods described for sentinel lymph node detection: vital stains and radioactive isotopes.

As for the first method, the three dyes most commonly used are isosulfan blue, patent blue violet, and methylene blue. The blue dye is injected around the tumor and 5–15 minutes after the injection the stain is localized in the draining lymphatics, which could remain coloured for up to 60 minutes. Injection of large volume of the dye into the tumor or intravascularly can produce high background signal intensity (shine effect) that could decrease the detection rates [18]. Another possible application problem could arise in large tumors, which are often centrally necrotic and this may cause retrograde leakage of the dye into the vagina through the cervical canal or needle penetration into the parametria with inadequate dye application. Some prerequisites such as careful preoperational identification of the residual stroma, utilization of a long spinal needle, and controlling of dye escape into the vagina or parametria can help in reaching higher detection rates [8, 19]. These water based blue dyes bind weakly to plasma proteins and are primarily excreted through the biliary tract. Adverse effects such as blue discoloration of urine, hypersensitivity reactions, and more rarely severe reactions have been reported when dye was mixed with local anesthetic agents [6].

The second method used includes the interstitial injection of radioactive materials.

99mTc-sulfur colloid is the material most commonly used in the United States, whereas 99mTc-nanocolloid human serum albumin is mostly used in Europe [20]. Dynamic scintigraphic imaging usually starts after the injection of the tracer for 20–30 minutes in order to reveal the progression of lymphatic flow and to determine the sentinel lymph node. A gamma probe is used to acquire the dynamic signal. The latter is moved slowly and carefully, so that the even small sentinel lymph nodes, in which the radiocolloid is retained in the sinusoidal spaces, are recognized. “Hot” nodes are identified in comparison with background radioactivity, which is defined as the average count rates of the surrounding nonsentinel nodes and lymph node basin [18]. The dose and the type of tracer injected, the elapsed time between tracer injection and surgery, and of course the type of gamma probe used influence the ratio values of sentinel lymph node counts to background counts. These ratios range [5] from 10 : 1 up to 25 : 1. Additional sentinel nodes are considered to have a counting rate at least 20% of the counting rate of the hottest node in the basin. Adequate sentinel lymph node excision is considered when residual radioactivity gets <10% of the counting rate in the hottest node [21].

The comparison between the two methods reveals that lymphoscintigraphy has several advantages over vital stains. First of all, it precisely locates the target sentinel nodes and guides accurately the surgeon during the dissection despite the possible presence of intraoperative bleeding. Harvest of the lymph nodes through a small incision is possible. Finally, it determines the possible presence of residual lymph nodes. On the other hand, it is more costly and time consuming and demands an accessible nuclear medicine unit with relevant safety protocols for operative handling of sentinel lymph node. In addition, the blue dye is especially useful in case of location of the sentinel lymph node in the parametrium, since the gamma probe count is influenced by the proximity of the cervix. The feasibility of sentinel lymph node identification in cervical carcinoma has been well documented with nearly 800 patients undergoing sentinel lymph node mapping in the reported literature. 81% is the pooled sensitivity using blue dye and 92% using technetium alone or combined technique. The detection rate for blue dye alone is 84% compared to 88% with technetium alone and 97% with the combined approach [22]. A sensitivity count of 100% and sentinel lymph node detection rate of 96% with the combined technique have also been reported in one of the largest studies [23]. The combined technique appears to be the most sensitive diagnostic technique for evaluation of the lymph node involvement in early cervical cancers compared with positron emission tomography, magnetic resonance imaging, and computed tomography [24]. Laparoscopic approach is a safe and accurate modality to identify and retrieve sentinel lymph nodes using both staining and radioisotopic techniques [25, 26]. This option allows shorter hospitalization and carries less morbidity than the open procedure [6]. In addition to the above, the visualization with the robotic laparoscopic 3D vision and magnification is thought to facilitate the identification of lymph nodes in general and nonsentinel lymph node tumour suspect nodes in particular adding extra accuracy to the SLN concept [27].

## 4. Beneficial Data from the Application of Sentinel Lymph Node Technique

A number of studies have been carried out in an attempt to further investigate the clinical usefulness of sentinel lymph node technique in cervical cancer patients. The most representative among them are mentioned below and their results are summarized in Table 1.

Roca et al. came to the conclusion that sentinel lymph node surgical biopsy based on blue dye and lymphoscintigraphy is a beneficial and useful technique in order to avoid lymph node dissection in the early stages of cervical cancer. As a result of its high negative predictive value and the simplicity of its incorporation into clinical routine (through laparoscopy or open surgery), this technique is close to achieving validation in this setting [28]. The combination of the sentinel node procedure and HPV DNA screening of the excised nodes is implied by another study in the direction of better evaluation of lymph node status and, hence, better identification of women who are at increased risk of recurrence [29]. A prospective multicenter study including 509 patients was driven by Altgassen et al. The authors concluded that systemic lymphadenectomy could not be omitted. The results of this study were however influenced by the inclusion of all stages, by the small number of procedures performed at each center, and by the definition of the detection rate. It is however mentioned that patients with tumor diameter < or = 20 mm may profit from this technique [30]. Darlin et al. indicate that the sentinel lymph node technique is probably an accurate method for the identification of lymph node metastases in cervical cancer patients with tumors of 2 cm or less. In this case, the negative predictive value reaches 100%, whereas in larger tumors it is 95%. According to their directions, a complete lymphadenectomy should be performed on the radionegative side in case of a unilateral sentinel lymph node only, and all bulky nodes must be removed [27]. Gortzak-Uzan et al. indicate that sentinel lymph node biopsy in early cervical cancer is a more sensitive procedure in detecting pelvic lymph node metastases compared to complete lymphadenectomy. False-negative and negative predictive values of 0% and 100%, respectively, were found [13]. It is additionally mentioned that the sentinel lymph node technique improves the detection rate of lymph node metastases by 2.8-fold [31]. This rate is found to be improved by SPECT/CT imaging which allows easier intraoperative detection with gamma probe as a result of better tridimensional anatomic location [32]. Du et al. revealed an association between sentinel lymph node detection rate and stage, tumor size, histologic type, preoperative treatment, and a history of preoperative conization. Their results demonstrated that sentinel lymph node biopsy is highly sensitive (100%) and accurate (100%) for diagnosing metastases in stages IA2–IB1 cervical cancer. The false-negative predictive value was 0% and the negative predictive value was 100%. Consequently, sentinel node navigation surgery in combination with careful preoperative evaluation for eligible patients and reliable intraoperative pathological investigation of sentinel nodes could provide large benefits for selected patients who desire fertility preservation [14]. Roy et al. nominate ultrastaging as the most important benefit of sentinel node mapping. Ultra-staging allows the identification of patients with micrometastases and isolated tumor cells in the nodes, elements, which predispose to recurrences [12]. Another potential benefit of sentinel lymph node mapping is also the reduction in the morbidity of the surgical management of early-stage cervical cancer. Reducing the radicality of parametrial resection for small tumor volume in sentinel lymph node negative patients is therefore both safe and feasible [33]. A recent study has revealed a detection rate of 97.5% (73.3% bilaterally) with no false-negative cases and no adverse reactions [34]. Cibula et al. in a large multicenter study observed a high sensitivity and a low false-negative rate of sentinel node staging when the sentinel nodes were detected bilaterally. It is proposed that sentinel node mapping and ultrastaging should become standard practice in the surgical management of early-stage cervical cancer [35].

Despite the great number of studies that have been carried out up to now, the critical question remains, whether sentinel lymph node biopsy can be incorporated in the everyday clinical practice.

## 5. Sentinel Lymph Node Navigation Surgery: Is It Ready for Routine Clinical Use?

So far, many studies support possible tailored treatment of cervical cancer by sentinel node navigation surgery, but there are still a few points to be elucidated before the introduction of this method in routine clinical practice.

First of all, a radioactive tracer is necessary in order to maintain a high detection rate, since the single method using blue dye only revealed relatively low detection rates compared with the dual method. Furthermore, the standardization of the method including the type, the volume, or the timing of the tracers should be accomplished before the routine use of the technique. Secondly, the omission of pelvic lymphadenectomy demands the presence of pathologists and speedy pathological diagnosis by intraoperative frozen sections. The risk of false-negative results remains another problematic issue. This risk is associated with the parametrial location of the sentinel node, the use of immunohistochemical techniques, the use of single method, and the application of the sentinel node identification procedure in bulky tumors or locally advanced stages with massive lymph node involvement [36]. As for the detection of sentinel nodes in the parametrium, it may be extremely difficult because of a shine-through phenomenon [37]. As far as locally advanced or bulky tumors are concerned, the higher incidence of missing metastasis (up to 20%) can be explained by the lymphatic obstruction by metastasis or inflammatory debris which alter the lymphatic drainage patterns [38, 39]. In addition to the above, bilateral detection of sentinel node is not always feasible; under these circumstances, complete lymphadenectomy on the nondetection side should be performed [27]. Finally, micrometastasis is an emerging issue associated with the possibility of recurrences. It is found in 15–43% of nodes identified as negative by immunohistochemical or reverse transcriptase-polymerase chain reaction [40–42].

The following algorithm proposed by Vicus and Covens can function as a guide in the sentinel lymph node evaluation. Injection of technetium takes place preoperatively in all four quadrants of the cervix submucosally. If at least one sentinel node is not apparent on each side of preoperative scintigram, then blue dye is injected submucosally in all four quadrants of the cervix when the patient is under anesthesia. In case of unilateral detection of sentinel lymph node, a complete pelvic lymphadenectomy should be performed on the nondetection side. Additionally, if macroscopically enlarged lymph nodes are noted intraoperatively, then the whole procedure should be abandoned and full lymphadenectomy should be performed [31]. A two-step strategy could also be an alternative option: at the first step, a systematic assessment of all sentinel lymph nodes including ultrastaging should be performed, followed by radical hysterectomy in case of negative nodes [43].

In conclusion, sentinel lymph node biopsy, without further pelvic lymphadenectomy, may be considered in patients with low-risk tumors, including tumors smaller than 2 cm, of grade 1 or 2, and of the most common histological subtypes such as squamous cell carcinoma (potentially effective radiation therapy), adenosquamous carcinoma, and adenocarcinoma [34].

## 6. Conclusions

Sentinel lymph node mapping has gained popularity in the gynaecological oncology, since it carries a higher detection rate of lymph node metastases and a much lower morbidity. Thus, further research through multicentre studies is needed in order to confirm the safety of omitting complete lymph node dissections in patients with negative sentinel lymph nodes and to surpass all the technical difficulties. In conclusion, a good team (consisting of both a surgeon and a nuclear medicine physician) is crucial in achieving the objectives of a higher detection rate of sentinel nodes, less aggressive operations, and, eventually, better clinical management.

## Figures and Tables

**Table 1:** Summary of clinical trials reviewed that served to validate the importance of sentinel lymph node mapping in cervical cancer patients.

Roca et al. [28]	Sentinel lymph node biopsy is a beneficial technique in order to avoid lymph node dissection in early stages of cervical cancer. It has a high negative predictive value and is simple to be incorporated into clinical routine.

Coutant et al. [29]	Combination of sentinel lymph node biopsy and HPV DNA screening of the excised nodes serves better evaluation of lymph node status and better identification of women at high risk of recurrence.

Altgassen et al. [30]	(i) Systemic lymphadenectomy cannot be omitted from the management of cervical cancer patients. (ii) Sentinel lymph node biopsy may be profitable for patients with tumour diameter < or = 20 mm.

Darlin et al. [27]	(i) The negative predictive value of sentinel lymph node biopsy reaches 100% in tumours < or = 20 mm and 95% in larger tumours. (ii) In case of unilateral sentinel lymph node detection, complete lymphadenectomy should be performed on the radionegative side. (iii) All bulky nodes should be removed.

Gortzak-Uzan et al. [13]	Sentinel lymph node biopsy is a more sensitive procedure in early cervical cancer compared to complete lymphadenectomy (false-negative value = 0%, negative predictive value = 100%).

Vicus and Covens [31]	Sentinel lymph node biopsy techniques improve the detection rate of lymph node metastases by 2.8-fold.

Díaz-Feijoo et al. [32]	SPECT/CT imaging improves the detection rate of lymph node metastases and allows easier intraoperative detection with gamma probe.

Du et al. [14]	(i) Sentinel lymph node biopsy is associated with stage, tumour size, histologic type, preoperative treatment, and history of preoperative conization. (ii) Sentinel lymph node biopsy has a sensitivity rate of 100%, an accuracy value of 100%, a false-negative predictive value of 0%, and a negative predictive value of 100% in stages IA2–IB1. (iii) Sentinel lymph node navigation surgery can provide large benefits for selected patients who desire fertility preservation.

Roy et al. [12]	Sentinel lymph node mapping in combination with ultrastaging can identify patients with micrometastases and isolated tumour cells in the nodes and eventually the patients with high risk of recurrence.

Diaz et al. [33]	(i) Sentinel lymph node mapping reduces the morbidity of surgical management of early-stage cervical cancer. (ii) Sentinel lymph node negative patients are safe to undergo a less radical parametrial resection for small tumour volume.

Devaja et al. [34]	Sentinel lymph node mapping techniques reach a detection rate of 97.5% (73.3% bilaterally), with no false-negative cases and no adverse reactions.

Cibula et al. [35]	(i) In cases of bilateral detection of sentinel nodes, the sentinel lymph node mapping method has a high sensitivity and a low false-negative rate. (ii) Sentinel lymph node mapping and ultrastaging should become standard practice in the surgical management of early-stage cervical cancer.

## References

[1] Parkin DM, Bray F, Ferlay J, Pisani P (2005). Global cancer statistics, 2002. *Ca-A Cancer Journal for Clinicians*.

[2] Jemal A, Bray F, Center MM, Ferlay J, Ward E, Forman D (2011). Global cancer statistics. *CA Cancer Journal for Clinicians*.

[3] Kokawa K, Takekida S, Kamiura S (2010). The incidence, treatment and prognosis of cervical carcinoma in young women: a retrospective analysis of 4,975 cases in Japan. *European Journal of Gynaecological Oncology*.

[4] Levenback C, Coleman RL, Burke TW, Bodurka-Bevers D, Wolf JK, Gershenson DM (2001). Intraoperative lymphatic mapping and sentinel node identification with blue dye in patients with vulvar cancer. *Gynecologic Oncology*.

[5] Levenback C (2008). Update on sentinel lymph node biopsy in gynecologic cancers. *Gynecologic Oncology*.

[6] Acharya BC, Jihong L (2009). Sentinel lymph node detection in patients with early cervical cancer. *Journal of the Nepal Medical Association*.

[7] Franchi M, Ghezzi F, Riva C, Miglierina M, Buttarelli M, Bolis P (2001). Postoperative complications after pelvic lymphadenectomy for the surgical staging of endometrial cancer. *Journal of Surgical Oncology*.

[8] Levenback C, Coleman RL, Burke TW (2002). Lympathic mapping and sentinel node identification in patients with cervix cancer undergoing radical hysterectomy and pelvic lymphadenectomy. *Journal of Clinical Oncology*.

[9] Matsuura Y, Kawagoe T, Toki N, Tanaka M, Kashimura M (2006). Long-standing complications after treatment for cancer of the uterine cervix—clinical significance of medical examination at 5 years after treatment. *International Journal of Gynecological Cancer*.

[10] Euscher ED, Malpica A, Atkinson EN, Levenback CF, Frumovitz M, Deavers MT (2008). Ultrastaging improves detection of metastases in sentinel lymph nodes of uterine cervix squamous cell carcinoma. *American Journal of Surgical Pathology*.

[11] Obnisbi S, Lomnes SJ, Laurence RG, Gogbasbian A, Mariant G, Frangioni JV (2005). Organic alternatives to quantum dots for intraoperative near-infrared fluorescent sentinel lymph node mapping. *Molecular Imaging*.

[12] Roy M, Bouchard-Fortier G, Popa I (2011). Value of sentinel node mapping in cancer of the cervix. *Gynecologic Oncology*.

[13] Gortzak-Uzan L, Jimenez W, Nofech-Mozes S (2010). Sentinel lymph node biopsy vs. pelvic lymphadenectomy in early stage cervical cancer: is it time to change the gold standard?. *Gynecologic Oncology*.

[14] Du X-L, Sheng X-G, Jiang T (2011). Sentinel lymph node biopsy as guidance for radical trachelectomy in young patients with early stage cervical cancer. *BMC Cancer*.

[15] Bilchik AJ, Giuliano A, Essner R (1998). Universal application of intraoperative lymphatic mapping and sentinel lymphadenectomy in solid neoplasms. *Cancer Journal from Scientific American*.

[16] Morton DL, Wen D-R, Wong JH (1992). Technical details of intraoperative lymphatic mapping for early stage melanoma. *Archives of Surgery*.

[17] Alex JC, Krag DN (1993). Gamma-probe guided localization of lymph nodes. *Surgical Oncology*.

[18] El-Ghobashy AE, Saidi SA (2009). Sentinel lymph node sampling in gynaecological cancers: techniques and clinical applications. *European Journal of Surgical Oncology*.

[19] Angioli R, Palaia I, Cipriani C (2005). Role of sentinel lymph node biopsy procedure in cervical cancer: a critical point of view. *Gynecologic Oncology*.

[20] Kapteijn BAE, Nieweg OE, Muller SH (1997). Validation of gamma probe detection of the sentinel node in melanoma. *Journal of Nuclear Medicine*.

[21] Mariani G, Gipponi M, Moresco L (2002). Radioguided sentinel lymph node biopsy in malignant cutaneous melanoma. *Journal of Nuclear Medicine*.

[22] van de Lande J, Torrenga B, Raijmakers PGHM (2007). Sentinel lymph node detection in early stage uterine cervix carcinoma: a systematic review. *Gynecologic Oncology*.

[23] Rob L, Strnad P, Robova H (2005). Study of lymphatic mapping and sentinel node identification in early stage cervical cancer. *Gynecologic Oncology*.

[24] Selman TJ, Mann C, Zamora J, Appleyard T-L, Khan K (2008). Diagnostic accuracy of tests for lymph node status in primary cervical cancer: a systematic review and meta-analysis. *Canadian Medical Association Journal*.

[25] Barranger E, Grahek D, Cortez A, Talbot JN, Uzan S, Darai E (2003). Laparoscopic sentinel lymph node procedure using a combination of patent blue and radioisotope in women with cervical carcinoma. *Cancer*.

[26] Martínez-Palones JM, Gil-Moreno A, Pérez-Benavente MA, Roca I, Xercavins J (2004). Intraoperative sentinel node identification in early stage cervical cancer using a combination of radiolabeled albumin injection and isosulfan blue dye injection. *Gynecologic Oncology*.

[27] Darlin L, Persson J, Bossmar T (2010). The sentinel node concept in early cervical cancer performs well in tumors smaller than 2 cm. *Gynecologic Oncology*.

[28] Roca I, Caresia AP, Gil-Moreno A (2005). Usefulness of sentinel lymph node detection in early stages of cervical cancer. *European Journal of Nuclear Medicine and Molecular Imaging*.

[29] Coutant C, Barranger E, Cortez A (2007). Frequency and prognostic significance of HPV DNA in sentinel lymph nodes of patients with cervical cancer. *Annals of Oncology*.

[30] Altgassen C, Hertel H, Brandstädt A, Köhler C, Dürst M, Schneider A (2008). Multicenter validation study of the sentinel lymph node concept in cervical cancer: AGO study group. *Journal of Clinical Oncology*.

[31] Vicus D, Covens A (2010). Role of sentinel lymph node biopsy in cervical cancer: pro. *International Journal of Gynecological Cancer*.

[32] Díaz-Feijoo B, Pérez-Benavente MA, Cabrera-Diaz S (2011). Change in clinical management of sentinel lymph node location in early stage cervical cancer: the role of SPECT/CT. *Gynecologic Oncology*.

[33] Diaz JP, Gemignani ML, Pandit-Taskar N (2011). Sentinel lymph node biopsy in the management of early-stage cervical carcinoma. *Gynecologic Oncology*.

[34] Devaja O, Mehra G, Coutts M (2012). A prospective single-center study of sentinel lymph node detection in cervical carcinoma. Is there a place in clinical practice?. *International Journal of Gynecological Cancer*.

[35] Cibula D, Cibula D, Abu-Rustum NR (2012). Bilateral ultrastaging of sentinel lymph node in cervical cancer: lowering the false-negative rate and improving the detection of micrometastasis. *Gynecologic Oncology*.

[36] Díaz-Feijoo B, Gil-Moreno A, Pérez-Benavente MA, Morchón S, Martínez-Palones JM, Xercavins J (2008). Sentinel lymph node identification and radical hysterectomy with lymphadenectomy in early stage cervical cancer: laparoscopy versus laparotomy. *Journal of Minimally Invasive Gynecology*.

[37] Covens A, Rosen B, Murphy J (2002). How important is removal of the parametrium at surgery for carcinoma of the cervix?. *Gynecologic Oncology*.

[38] Slama J, Dundr P, Dusek L (2012). Sentinel lymph node status in patients with locally advanced cervical cancers and impact of neoadjuvant chemotherapy. *Gynecologic Oncology*.

[39] van Oostrum NHM, Makar APH, van den Broecke R (2012). Sentinel node procedures in gynecologic cancers: an overview. *Acta Obstetricia et Gynecologica Scandinavica*.

[40] Lentz SE, Muderspach LI, Felix JC, Ye W, Groshen S, Amezcua CA (2004). Identification of micrometastases in histologically negative lymph nodes of early-stage cervical cancer patients. *Obstetrics and Gynecology*.

[41] Wang HY, Sun JM, Lu HF (2006). Micrometastases detected by cytokeratin 19 expression in sentinel lymph nodes of patients with early-stage cervical cancer. *International Journal of Gynecological Cancer*.

[42] Daraï E, Rouzier R, Ballester M, Barranger E, Coutant C (2008). Sentinel lymph node biopsy in gynaecological cancers: the importance of micrometastases in cervical cancer. *Surgical Oncology*.

[43] Bats A-S, Buénerd A, Querleu D (2011). Diagnostic value of intraoperative examination of sentinel lymph node in early cervical cancer: a prospective, multicenter study. *Gynecologic Oncology*.

